# Alcoholism with Suggestions as to Treatment—Statistics for Buffalo1Read before the Buffalo Academy of Medicine.

**Published:** 1895-10

**Authors:** Sidney A. Dunham

**Affiliations:** Buffalo, N. Y., Physician to Lexington Heights Hospital, Department of Alcoholism and Dipsomania


### ALCOHOLISM WITH SUGGESTIONS AS TO TREATMENT —STATISTICS FOR BUFFALO.[1]

By SIDNEY A. DUNHAM, M. D., Buffalo, N. Y.,
Physician to Lexington Heights Hospital, Department of Alcoholism and Dipsomania.

FROM the police reports we find from 1890 to 1894, inclusive, that there were 33,909 arrests for drunkenness in Buffalo. Of this number of arrests 29,895 were men, 4,014 were women, or an average for each month of 498 men and sixty-six women. During the same period of five years there were 16,213 arrests for disorderly conduct. Of this number 13,480 were men, 2,733 were women, or an average for each month of 224 men and forty-five women. Of those classed under this head, it is safe to say that the majority were drunk as well as disorderly. During the same period 490 were classed under the head of dipsomaniacs and sent to the jail to sober up. The Erie County Penitentiary reports for the five years show that 16,949 men were there for drunkenness and 3,080 women were sent to the same place. While more than one-half of the arrests for drunkenness were sent to the penitentiary, about one-third of those arrested for disorderly conduct were sent also to the penitentiary. One man has a history of being sent to the penitentiary for drunkenness fourteen times in one year and another thirteen times. Of those sent to the jail for dipsomania nineteen were in twice and nine three times.

During this period of five years, reports from the coroner's office show that seventy-nine died from alcoholism, and out of the 221 suicides seventy-three were known to have been hard drinkers. According to French authorities, the preponderate cause of suicide is alcoholism. The reports quoted show that nearly half of the suicides in the city are due, directly or indirectly, to alcoholism ; and if a correct history of these cases could always be obtained, there is no doubt that the majority of suicides would be found of that class.

Comparing the small number treated at hospitals, where drink was the cause of their confinement, with those that were retained for punishment, we find that only 264 men and forty-three women were in the Buffalo General Hospital during those five years, and that 169 men and thirty-nine women were at the Buffalo State Hospital or asylum. Probably an equal number were treated at the Sisters' of Charity Hospital. However, the reports show a

1. Read before the Buffalo Academy of Medicine.

very small number receiving treatment for a disease which held these habituates as slaves to appetite.

Dealing with it as a crime against law and order, severe fines and heavy punishments have been applied in all ages. But a very small percentage have been benefited of the large number of those thus treated. Treating it as a disease, the last decade has revolutionised the theories as to the cause, also care, of such maladies, and the progress has been as great in this direction as that which has marked the treatment of many other diseases. Experience teaches us that it is a disease which can be successfully treated, the time and course of treatment depending upon the stage and character of the disease. The treatment of this class of patients has varied from the heroic method of blood-letting in olden times to the empiric method of hypnotic suggestion of the present day.

Like other diseases, dipsomania or chronic alcoholism may be inherited or acquired. In either case the treatment is the same, although the results may be different. The more chronic the case, the more persistent should be the treatment. Habit leads to disease just as exposure leads to rheumatism. The strong-nerved man finds appetite often more than he can overcome. Therefore, we should expect that the one who has inherited a nervous weakness should frequently find accustomed appetites uncontrollable.

Heredity predisposes to dipsomania, the same as it predisposes to consumption. Both are developed under favorable surroundings. Asthma is a neurotic disease, inherited, but there is a climate for every asthmatic. Also, there is a climate for every hard drinker (temperate climate).

*How They are Treated at Present.*—Medically, the patient is confined to his room, either because he is unable to go out of his own accord or because he is constrained by friends. The physician prescribes medicines to overcome the alarming symptoms, such as uneasiness and sleeplessness, together with a steady withdrawal of stimulants. As soon as the patient talks rationally and walks steadily the physician discharges his patient or the patient discharges his physician. This course of treatment lasts from three days to one week, and is the sum of the entire medical attention the patient receives when he has been suffering from a chronic disease for several years. We need not be surprised that relapses occur very shortly and frequently. No other disease, so grave in character, so dangerous to the community and fatal to the patient, is today treated so carelessly and unscientifically.

Legally, the misdemenant is arrested, spends a night in a cell at the station-house and is fined $5.00 to $10.00 in the morning or an equal number of days at the workhouse. If he be well connected, socially and financially, he may be detained in the jail until his physical and mental condition is sufficiently improved to warrant the authorities in discharging him on the promise that it will be "the last time." When the patient is given an opportunity to go out into the world to do better, it is only a short time ere he finds himself again overcome by his tempter. In those cases where drunkenness is a vice, this treatment is insufficient; and in those where it is a disease, it is cruel and barbarous.

*Why They Should be Differently Treated.*—Because a careful physical examination reveals lessened nervous energy, weakened cerebral functions, interference with coördination and reflex action, altered secretions and diminished excretions. And further, because a mental examination reveals hallucinations (painful or pleasant), delusions, melancholia, suicidal tendencies, and the like, and shows weakened will power, temporary loss of memory, the finer sensibilities numbed, perceptions and emotions dulled, truth, decency, duty, honor and felicity doubtful or altogether lost. In every case there is evidence of the paralysing effect of alcohol upon the inhibiting power of the brain, which is one of its highest faculties; and herein the greatest damage is wrought and the will undermined.

Again, because the pathology of this disease, in advanced stages, shows degeneration of the nerve tissue, hyperemia of the meninges of the brain, diffused interstitial sclerosis of the cord, with cirrhotic changes of the internal organs.

Charpentier, out of 135 victims of general paresis, found eighty three cases were confirmed alcoholics. He adds alcohol to syphilis and heredity and calls them the triad of general paresis. As in severe forms of indigestion, with altered secretion and functional disturbances, when the *post mortem* shows no pathological changes, so in severe and dangerous forms of alcoholism, with perverted faculties and impulses, the brain itself may reveal no structural changes. Sonderegger considers drunkenness the effect and not the cause of the disease; that it is an irregular development and distribution of the cells through which the will and conscience act.

There are premonitory symptoms in periodic drinkers, such as nervousness, irritable disposition, forgetfulness, deep meditation, poor appetite and sleeplessness. Baker (*Boston Medical and Sur-*

*gical Journal*) reports a case of hereditary dipsomania, in which the patient, during his craving for alcohol, though prevented from getting it, would become sleepless, lose his appetite, appear silly, incoherent, staggering in gait, with some delusions of persecution manifested. As a patient expressed it to me the other day: "I am in another world during the drinking period."

*How They Should be Treated.*—From a criminal standpoint, the penalties should be multiplied by ten. It should be 100 days' imprisonment where it is now only ten days. This would give the patient an idea of the gravity of the offense and will also give Nature time in which to gain strength and fortify herself against future temptations and indulgence. While thus restrained, the patient should be kept at light work and receive medical aid for, at least, four weeks, according to the most advanced and successful methods of the present day.

The medical treatment, where it is now continued for about five days on the average, is about one-tenth the time required to materially benefit these cases. The indications to be met generally are largely a disturbance of the nervous system, which manifest themselves in an irritable disposition, sleeplessness, fits of depression, and, later, excitability, with all the phenomena of mania-a-potu. The digestive processes are sluggish and weak in character, the excretions are deficient and there is a general loss of muscular tone.

The excitable stages are best controlled by chloral and bromides; or, when there is much delirium with a strong pulse, hypodermic injections of hydrobromate of hyoscine 1-100th to 1-150th of a grain, to be repeated in six hours, or smaller doses every four hours. Ergotin, added in small doses to the above, will overcome the unpleasant effects of hyoscine with a good result on the cerebral congestion. Stimulants should be rapidly withdrawn, and when given should be administered in milk and other foods, but never should be given clear or "straight." If there is much nausea, it can be controlled by small doses of calomel and bismuth, frequently repeated, with nourishment given a little at a time and often. As soon as the patient is able to take nourishment, it should be fluid in character and large in quantity, highly seasoned. If the bowels are constipated, they should be opened by injections of water and glycerine, when the patient is not able to take alkaline aperients through the stomach in the ordinary manner. An infusion of digitalis, tablespoonful doses, with ten grains of citrate of potash every four hours, will increase the urinary excretions when they are diminished. Hypodermics of the nitrate or sulphate of strychnine, with a little digitalin, are the best to overcome heart weakness and often relieves the delirium. Mulford's tablets for dipsomania for hypodermic use will be found very serviceable—namely, gold and sodium chloride, 1-24 gr.; strychnine nitrate, 1-60 gr.; nitroglycerin, 1-300 gr.; atropine sulph., 1-200 gr.; digitalin, 1-60 gr.; sodium chloride, $\frac{1}{8}$ gr.

Cold to the head overcomes delirium when due to congestion or active hyperemia of the meninges.

When the acute symptoms have subsided, a three or four weeks' course of curative treatment should begin. A tonic, consisting of nux vomica, hydrastis, capsicum and an infusion of gentian, should be given four times a day and in full doses. Also hypodermics of the chloride of gold in solution—one-tenth of a grain to ten minims of distilled water—should be given three or four times a day.

Tablets of the above formula are good for the first week or ten days, followed by the gold solution. The platinum needle will not corrode by the gold solution and should be used for this reason. The infusion of gentian is used because it contains less alcohol than the tincture.

Because the chloride of gold meets so many indications in the treatment of dipsomania, there is no more reason to call it a "gold cure for dipsomania" than there is to call it a gold cure for consumption, where it has been used with a certain degree of success; or a "gold cure" for rheumatism with deformed joints, where it has been found valuable; or a "gold cure" for paralysis of the insane, where it is one of the most efficacious remedies.

The chloride of gold I consider one of the chief therapeutic agents in the treatment of chronic alcoholism. It has been used in cases of melancholia, hysteria, chorea, and especially nervous troubles due to syphilis. In its physiological action it seems to be a tonic for the brain and spinal cord—an alterative like mercury; it stimulates nutrition and digestion, increases secretions and excretions, and is an aphrodisiac. Its action in this respect is like that of strychnine and phosphorus.

The belief that the impotency for a time following the treatment is due to the chloride of gold, is a delusion on the part of the patient.

Strychnine tones up the nerve centers and the walls of arteries are able to contract to the normal caliber, while muscular fibers return to their healthful response.

If the alcoholic should require both punishment and medicine, as they usually do, the hypodermic method, four times a day, meets the indication beautifully. If the arms swell from the effects of the hypodermics, Goulard's extract will overcome the difficulty.

In some cases, craving for drink can only be removed by treating physical ailments. Lawson Tait reports cases of cure following the removal of the uterine appendages in women. Indigestion is to be treated, neuralgia and nervous exhaustion to be remedied, irregular and weak heart action to be overcome, environment and habits to be changed, syphilis and kidney trouble to receive attention. Irregular hours at meals and for sleep, the futile attempt to drink moderately, old associations in drinking, the intention to drink only beer and cider, all predispose to alcoholic excess in those already habituated to excessive drinking.

Some believe in inebriate asylums under the control of the state. Our insane asylums are just as good, and this class of patients go as willingly to the latter as to the former. The difficulty, at present, with our hospitals is their having no power to hold these patients as long as is required for successful treatment. Every city of the size of Buffalo, showing the amount of alcoholism that Buffalo does, is in need of a hospital for inebriates, endowed with power to retain patients until dismissed as cured. Our present overcrowded penitentiary would be greatly relieved of some of its very heavy burdens, the community better protected and the reformation and cure of the victim would be more pronounced and permanent. I agree with Dr. T. B. Crothers, of Hartford, that public sentiment should not permit one to become an inebriate, or tolerate him after that stage, unless under legal guardianship and restriction, until he recovers.

Our morning justices should impose larger fines for drunkenness, which means longer terms of confinement. Medical treatment should be enforced for not less than four weeks while serving time. Our habitual drinkers who do not voluntarily take treatment should be committed to the asylum until a more suitable institution can be established. The state should arrange for the proper treatment of these cases when in penal institutions. A law is required to meet this end. Every municipality should have this done.

Drunkenness, with beginners, is of so great moment, because of its demoralising effect upon youth, that, to be a preventive, fines must be large in order to be commensurate with the offense. While there may be a question about the responsibility of acts committed while under the influence of liquor, there is no doubt about the great responsibility of such persons getting drunk as a beginner.

A portion of mankind have proven in the past that they could not drink moderately, and ought not, therefore, to have taken liquor at all, as the first drinks (socially, it may be,) developed the uncontrollable desire which previous to that time had been latent and ought never to have been cultivated.

The moderate drinkers of today are going to furnish us with the dipsomaniacs tomorrow.

Those who have inherited a predisposition to consumption would not hazard their lives by continually nursing and associating with the consumptive. This applies to all those who have inherited irritable, nervous systems and thereby are predisposed to drink.

It is almost impossible to carry out this system of treatment in private practice. It can be more thoroughly done and the psychical effect upon the patient far more beneficial when sent to an institution. The Lexington Heights Hospital of this city has arranged particularly for these cases, and when treated according to this method the results are very satisfactory. Those who manifest a desire to take treatment and admit that they are unable themselves to discontinue the use of liquor, give the highest percentage of cures. Those who receive the least benefit remind one, when asked why they do not take treatment, of Rogers's lines :

"Go ! you may call it madness, folly,
You shall not chase my grief away ;
There's such a joy in melancholy
I would not, if I could, be gay."

The report of two prominent French medical authorities, Brouardel and Bouchet, to the French hygienic government committee, declare that the future belongs to abstemious nations ; that it is not only a social danger everywhere, but that the body and mind of posterity are weakened. My object in presenting this paper is not to stir your emotions and entertain your higher sensibilities for the present, but to appeal to your intellectual, scientific and executive powers for the better treatment and control, in the future, of these victims of habit and disease.

72 West Chippewa Street.

